# VIRTUAL GERAS DANCING FOR COGNITION EXERCISE (DANCE) FOR OLDER ADULTS: A FEASIBILITY RANDOMIZED CONTROL TRIAL

**DOI:** 10.1093/geroni/igad104.1981

**Published:** 2023-12-21

**Authors:** Patricia Hewston, George Ioannidis, Courtney Kennedy, Alexandra Papaioannou

**Affiliations:** McMaster University, Hamilton, Ontario, Canada; GERAS Centre for Aging Research, Hamilton, Ontario, Canada; McMaster University, Hamilton, Ontario, Canada; McMaster University, Hamilton, Ontario, Canada

## Abstract

**Background:**

GERAS DANcing for Cognition and Exercise (DANCE) was developed with rehabilitation and geriatric medicine expertise for older adults (age 60+) looking to improve brain health or mobility. Our primary aim of this study was to assess the feasibility of virtual GERAS DANCE.

**Methods:**

This study utilized a single-center, prospective, parallel-group randomized controlled trial (RCT) feasibility approach. We recruited 50 older adults. Participants were randomized to receive 6-weeks (1-hour class twice weekly) of virtual GERAS DANCE or usual care. Feasibility was assessed using pre-defined criteria for process, outcomes, and acceptability, and the effect of GERAS DANCE on mood, balance confidence, and fear of falling.

**Results:**

Our study recruitment period occurred over 8 weeks to recruit 50 older adults (mean age = 75.02(5.89) years, range: 63-92, 92% female). The enrollment-to-screening ratio was calculated as 25:103 and the retention rate of participants was 84%. The average class attendance of the study cohort was 77%. One adverse event was reported unrelated to the study intervention. The program had a high-fidelity score and adhered to the standardized curriculum. Both the intervention and usual care groups improved mood (Depression, Anxiety and Stress Scale – 21). The intervention-group had higher balance confidence and lower fear of falling.

**Discussion:**

Pre-determined thresholds for feasibility were met for all outcomes providing evidence that virtual GERAS DANCE is feasible, well-accepted, and safe for older adults. Improved balance confidence and reduced fear of falling through dance can have significant implications for fall prevention, healthcare costs, and overall quality of life.

